# Effect of Bone Marrow Mesenchymal Stromal Cell Therapies in Rodent Models of Sepsis: A Meta-Analysis

**DOI:** 10.3389/fimmu.2021.792098

**Published:** 2022-01-03

**Authors:** Lite Ge, Jing Zhao, Huiyin Deng, Chunli Chen, Zhiping Hu, Liuwang Zeng

**Affiliations:** ^1^ Department of Neurology, Second Xiangya Hospital, Central South University, Changsha, China; ^2^ Hunan Provincical Key Laboratory of Neurorestoratology, The Second Affiliated Hospital, Hunan Normal University, Changsha, China; ^3^ Department of Anesthesiology, The Third Xiangya Hospital, Central South University, Changsha, China

**Keywords:** sepsis, meta-analysis, efficacy, animal experimentation, bone marrow stromal cell (BMSC)

## Abstract

**Background:**

Multiple preclinical studies have demonstrated that bone‐marrow derived mesenchymal stromal (stem) cells [MSC(M)] positively influence the severity of sepsis symptoms and mortality in rodent models. However, this remains an inconclusive finding.

**Objective:**

To review the effect of naïve MSC(M) in rodent models of sepsis.

**Methods:**

The PubMed, EMBASE, and Web of Science databases were searched up to August 31, 2021. Inclusion criteria according to PICOS criteria were as follows: (1) population: rodents; (2) intervention: unmodified MSC(M); (3) comparison: not specified; (4) primary outcome: the effects of MSC(M) cell therapy on the mortality of rodent models of sepsis and endotoxemia; (5) study: experimental studies. Multiple prespecified subgroup and meta-regression analysis were conducted. Following quality assessment, random effects models were used for this meta-analysis.The inverse variance method of the fixed effects model was used to calculate the pooled odds ratios (ORs) and their 95% confidence intervals (CIs).

**Results:**

twenty-four animal studies met the inclusion criteria. Our results revealed an overall OR difference between animals treated with naïve MSC(M) and controls for mortality rate was 0.34(95% confidence interval: 0.27-0.44; P < 0.0001). Significant heterogeneity among studies was observed.

**Conclusions:**

The findings of this meta-analysis suggest that naïve MSC(M) therapy decreased mortality in rodent models of sepsis. Additionally, we identified several key knowledge gaps, including the lack of large animal studies and uncertainty regarding the optimal dose of MSC(M) transplantation in sepsis. Before MSC(M) treatment can advance to clinical trials, these knowledge gaps must be addressed.

## Introduction

A potentially lethal disease involving multiple systems, sepsis is caused by an abnormal balance between the host’s proinflammatory response and antiinflammatory response to infection (1, 2). Approximately 40% of deaths in the intensive care unit (ICU) are caused by sepsis, one of the principal causes of mortality worldwide (3, 4). The development of treatments for sepsis requires substantial advances in understanding of the diagnostic mechanisms and regulatory mechanisms that modulate the host’s response to infecting organisms. Despite substantial progress in understanding, there remain significant challenges in translating these advances into clinically effective therapies (5, 6). Presently, no specific treatment is available for sepsis and septic shock. Only symptomatic management is available, which includes the infusion of antibiotics and catecholamines (7). It appears that cellular therapy may offer great potential for the treatment of sepsis and septic shock. There are a number of different source tissues that can be used to treat sepsis, such as human bone marrow, adipose, olfactory mucosa, placenta, umbilical cord, and umbilical cord blood (8). Among the different kinds of mesenchymal stem cells that have been studied in preclinical studies, bone marrow derived mesenchymal stem cells have been thoroughly tested.

Bone marrow mesenchymal stromal cells (MSC(M), also known as bone marrow stem cells, or bone marrow-derived stem and progenitor cells) were firstly described by Freidenstein et al. in 1968 (9). They were adherent, fibroblast-like clonogenic cells with a high capability of replicating and differentiating into osteoblasts and adipocytes (10, 11). Cells in the bone marrow comprise less than 0.1% of mesenchymal stromal cells. However, MSC(M) can be harvested and expanded easily in culture, which makes them attractive for research. Additionally, allogeneic therapy is also available. It is because of these characteristics that MSCs are attractive for clinical trials. The feasibility and efficacy of mesenchymal stem-cell-based therapy has been examined in a large number of clinical trials (12, 13). It is estimated that over 2000 patients have benefited from over 400 clinical trials using MSCs to treat graft-versus-host disease, diabetes, hematological malignancies, cardiovascular disease, neurologic diseases, and autoimmune diseases.

MSC(M) may hold the key for sepsis and are therefore an appealing reparative therapy. There is now a growing body of preclinical evidence investigating the efficacy of MSC(M) therapy (14–36); however, there are often conflicting results in the literature. Güldner et al. found that the survival rate of mice did not differ among treated with mouse MSC(M), human MSC(M),and the untreated animals (CLP) (26). A similar result was also published by Silva et al. In their study there was no statistical difference in survival rates between the MSC(M) and CLP groups (34). Prior meta-analysis either took a broader approach to mesenchymal stem cells therapy or database was only until October 1, 2017 (37, 38). Neither have offered a meta-analysis of the relevant study to investigate mortality rate of MSC(M) transplantation for sepsis. Our aim was to perform a meta-analysis to review published animal studies employing the use of naïve MSC(M) therapy following sepsis, and to provide information for the future clinical translation of MSC(M) to the bedside.

## Materials and Methods

In accordance with Preferred Reporting Items for Systematic Reviews and Meta-Analyses (PRISMA) (39), a meta-analysis has been conducted. Analyzing data that is available in published articles does not require ethical approval or consent from the patient. The article provides all supporting data and the online supplement provides additional information

### Search Strategy

The researchers conducted a systematic literature search using 3 databases, including MEDLINE, EMBASE, and Web of Science, to screen for targeted studies on the efficacy of BMSCs in treating sepsis. The detailed search strategy is shown in Additional file 1: **Table S1**. The last search was updated on August 31, 2021. The publication language was limited to English. We also searched the reference lists of eligible studies.

### Inclusion and Exclusion Criteria

According to the PICOS scheme (population, intervention, control, outcome, and study design), the studies’ eligibility criteria were formulated (40). This meta-analysis included studies that met the following criteria: (i) They tested the efficacy of MSC(M) treatment in animal models of sepsis (all types of animals and both genders). (ii) The research centered on animal models of sepsis or endotoxemia. (iii) The study included mortality as part of its evaluation index. When two or more articles contained overlapping data, the most recent or informative article was used. (iv) Present experimental studies in original research papers. (v) The study was published in English.

The exclusion criteria were as follows: (i) Researches that only evaluated the efficacy of transfected or modified cell transplantation. (ii) Studies that only tested stem cells other than MSC(M). (iii) MSC(M) administered before sepsis model.

### Study Selection

All published articles were independently reviewed by two investigators after the removal of duplicates. After the two investigators reached an agreement, irrelevant studies were excluded. For a comprehensive review, all relevant articles were retrieved, and two researchers independently evaluated each article using the above selection criteria. Any disagreements or uncertainties were resolved by consensus, and if necessary by a third investigator.

### Data Abstraction

The following information were abstracted by two investigators independently and entered electronically: author, publishing year, study country, animal characteristics (species, gender, sample size, and model), intervention characteristics (origin, dose, route, and timing of the MSC(M) treatment), follow-up (the longest observation time of outcomes after MSC(M) administration), and our primary measures related to secondary outcomes. If only graphs were available, values were calculated from images using GetData Graph Digitizer software. Analyzing the data involved averaging the two researchers’ readings. In the absence of the standard deviation, the standard error was converted into a standard deviation by multiplying it by the square root of the group size. When multiple experimental groups that were distinguished by a variety of factors such as cell dose or delivery route and timing were contrasted with the control group, these groups were considered separate and independent studies. When results were evaluated over a range of follow-up periods, only the longest interval was selected.

### Risk of Bias (ROB)

Study authors assessed independently the risk of bias for each study included using the method developed by the Systematic Review Center for Laboratory Animal Experimentatio(SYRCLE) (41). This tool evaluates selection bias, performance bias, detection bias, attribution bias, and reporting bias and grades them according to “Yes, No, or Unclear.” Arguments were resolved through discussions with additional authors.

### Statistical Analysis

The primary outcome of this meta-analysis was mortality. OR was calculated between the MSC(M) treated group and the control group to determine the combined effect size. All statistical analyses and graphs were performed by R (version 4.0.2), using a random-effects model and the Hedges calculation (42). Typically, an effect size of 0.2 represents a small effect, 0.5 represents a medium effect, and 0.8 represents a large effect (43). A P-value <0.05 was considered statistically significant. The *I* ^2^ statistic was used to analyze heterogeneity, and it was divided into three categories, low (25–50%), moderate (50–75%), and high (>75%) (44).

We used six clinical characteristics to group the effect size of outcome: Gender (male, female); sepsis model (CLP, non-CLP Roteneone); MSC(M) species (Allogeneic, Syngeneic, or Xenogeneic); MSC(M) dose (<1×10^6^, ≥1×10^6^ and <1×10^7^, ≥1×10^7^); follow-up duration (≤6hours, >6 hours); delivery route (intravenous or intraperitoneal). In order to examine the possible associations between the outcomes and the above clinical characteristics, subgroup analyses and meta-regression analyses were performed (45). Sensitivity analyses were performed by omitting one study at a time to evaluate whether the results were affected by a single study. Publication bias was evaluated using funnel plots (46), and the symmetry of funnel plots was performed with Egger regression (47). If necessary, any non-negligible bias would be corrected using the trim-and-fill approach (48).

## Results

### Study Inclusion

**Figure 1:**
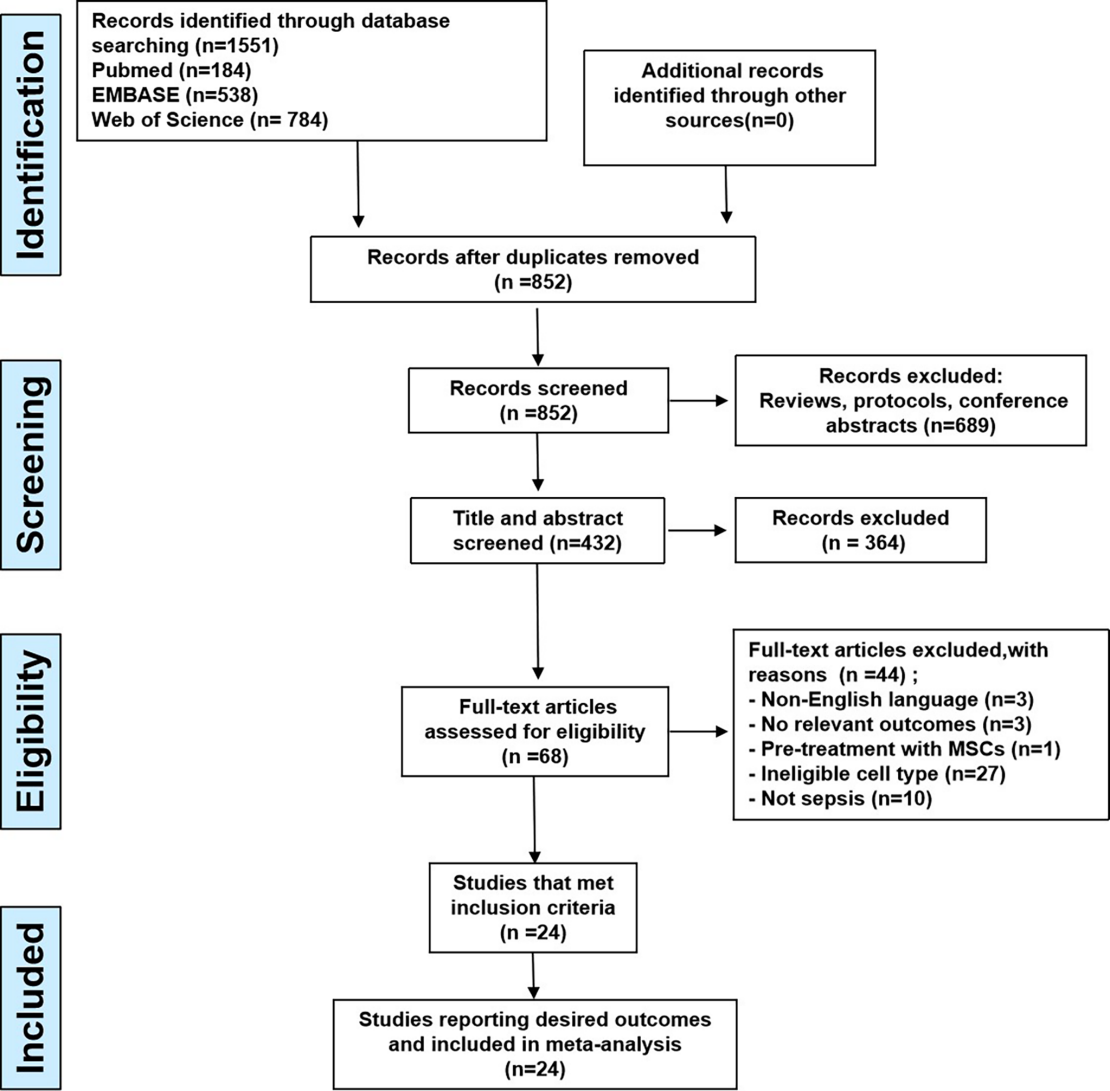
The flow diagram describing literature search and study selection.

A total of 1551 records were identified following the search procedure shown in **Figure 1**, including 184 articles in PubMed, 538 articles in EMBASE, and 784 articles in Web of Science. After excluding duplicates, 432 studies were screened by title and abstract and 364 studies were subsequently excluded. Full-text screening for eligibility was performed on 68 studies and, based on the inclusion and exclusion criteria, 24 studies were included in this systematic review, No new records met our inclusion criteria, and thus, all were excluded.

### Study Characteristics

The characteristics of the 24 studies are summarized in **Table 1**. All studies were carried out in rodents (rats and mice). Intervention included MSCs obtained from mice, rat, or human bone marrow. The most common sepsis models were the cecal ligation and puncture (CLP) and intraperitoneal injection of lipopolysaccharide (LPS). Following induction of sepsis, MSC(M) were injected within a few hours of the induction of the sepsis animal models. The mean follow-up ranged from 8 days to 20 weeks. The most common delivery routes used for MSC(M) were intravenous route or intraperitoneal route. The total dose of MSC(M) administered ranged from 1×10^5^ to 1×10^7^ cells.

**Table 1:** Characteristics of included studies.

Year	Author	Country	Species	Strain	Gender	No. of Treated Animals	No. of Controls	Sepsis Model	BMSC Species	Compatibility MSC Dose	Time of Delivery Post-sepsis Induction	MSC route	Control
2009	Nemeth et al. (14)	USA	mouse	C57BL/6	Male	90	45	CLP	Allogeneic	1.0×10^6^	0 or 1 hour	IV	PBS
2010	Bi et al. (15)	China	mouse	C57BL/6	Not Reported	10	10	CLP	Xenogenic	1.0×10^6^	1 1 hours	IV	PBS
2010	Mei et al (16)	Canada	mouse	C57BL/6	Female	29	29	CLP	Syngeneic	2.5×10^5^	6 hours	IV	NS
2011	Mei et al. (16)	Canada	mouse	C57BL/6	Female	15	20	CLP	Syngeneic	2.5×10^5^	6 hours	IV	NS
2012	Liang et al. (17)	China	rat	Wistar	Female	15	15	LPS (i.v.)	Syngeneic	1.0×10^6^	2 hours	IV	NS
2013	Krasnodembskaya et al. (18)	USA	mouse	C57BL/6	Male	34	69	P. aeruginosa(i.p.)	Xenogenic	1.0×10^6^	1 hour	IV	PBS
2013	Hall et al. (19)	USA	mouse	BALB/c	Male	26	35	CLP	Syngeneic	1× 5.0 ×10^5^ + 2 ×2.5×10^5^	2 then 24 then 48 hours	IV	PBS
2013	Zhao et al. (20)	China	rat	SPD	Female	24	27	LPS (i.v.)	Syngeneic	2.5×10^6^	2 hours	IV	NS
2014	Chao et al. (21)	Taiwan(China)	rat	Wistar	Male	20	10	CLP	Xenogenic	5.0×10^6^	4 hours	IV	PBS
2014	Kim et al. (22)	Canada	mouse	C57Bl/6	Male	73	66	SEB+ (i.p)	Syngeneic	2.5×10^5^	3 hours	IV	PBS
2014	Luo et al. (23)	China	mouse	C57Bl/6	Male	20	20	CLP	Syngeneic	1.0×10^6^	3 hours	IV	NS
2014	Sepulveda et al. (24)	Spain	mouse	BALB/c	Male	30	10	LPS (i.p.)	Xenogenic	1.0×10^6^	0.5 hour	IP	PBS
2015	Hao Ou et al. (25)	China	mouse	SPF	Not Reported	9	14	LPS	Allogeneic	1.0×10^7^	5 mins	IV	NS
2015	Güldner et al. (26)	Brazil	mouse	BALB/c	Not Reported	32	29	CLP	Xenogenic	1×10^5^	24 hours	IV	NS
2015	Güldner et al. (26)	Brazil	mouse	BALB/c	Not Reported	35	29	CLP	Allogeneic	1×10^5^	24 hours	IV	NS
2016	Liu et al. (27)	China	mouse	C57BL/6	Male	20	20	CLP	Allogeneic	1×10^6^	0 hours	IV	NS
2017	Xu et al. (28)	China	mouse	C57BL/6	Male	12	12	CLP	Allogeneic	1×10^6^	6 hours	IV	PBS
2018	Li et al. (29)	China	mouse	C57BL/6	Not Reported	20	20	CLP	Allogeneic	2.5×10^5^	24 hours	IV	NS
2018	Saeedi et al. (30)	Iran	mouse	C57BL/6	Male	10	10	E coli	Xenogenic	1×10^6^	16 hours	IV	NS
2018	Saeedi et al. (31)	Iran	mouse	C57BL/6	Male	10	10	LPS	Syngeneic	1×10^6^	16 hours	IV	PBS
2019	Laroye et al. (32)	France	mouse	C57BL/6	Male	48	48	CLP	Xenogenic	2.5×10^5^	24 hours	IV	PBS
2020	Luo et al. (33)	China	mouse	C57BL/6	Male	20	20	CLP	Allogeneic	1×10^6^	3 hours	IV	NS
2020	Silva et al. (34)	Brazil	mouse	Swiss Webster	Not Reported	30	30	CLP	Allogeneic	1×10^5^	6 hours	IV	NS
2021	Varkouhi et al. (35)	Canada	mouse	C57BL/6	Male	17	19	CLP	Xenogenic	2.5×10^5^	6 hours	IV	PBS
2021	Guo et al. (36)	China	rat	SPD	Famale	6	6	CLP	Syngeneic	1×10^6^	3 hours	IV	PBS

CLP, Cecal ligation and puncture; E. coli, Escherichia coli; IP, Intraperitoneal; IV, Intravenous; LPS Lipopolysaccharide; NR, Not reported; NS, Normal saline; P. aeruginosa, Pseudomonas aeruginosa; PBS, Phosphate buffered saline; SEB, Staphylococcal enterotoxin B; SPD, Sprague Dawley; SPF, Specific pathogen free.

### Assessment of RoB

The RoB assessment results of included studies is summarized in **Table 2**. There was no study considered to have a low RoB. There was no significant difference in the baseline characteristics between the experimental and control groups, reducing the chance of selection bias based on animal characteristics. No study has explicitly described how random sequences are generated despite the random allocation of experimental and control subjects. Accordingly, the RoB was deemed to be “unclear” in the sequence generation domain of all studies included. However, no study attempted to describe the method of concealed allocation. An evaluation of randomized outcomes was reported, the intervention received by each animal was blinded to researchers and, in one study (22), the evaluator’s blindness was noted. Based on the provided signal questions, all of the included studies showed a low risk of attrition bias and reporting bias. In one study that had a high risk of bias, the decrease in animal numbers was not accounted for between methods and results (15). In one study (16), there were other problems that caused a high risk of bias, such as pollution, experimental design, etc. Furthermore, we did not identify any additional sources of bias or bias tools in relation to systematic risks that were not included.

**Table 2:** SYRCLE Risk of Bias Assessment for included studies.

Author (Year)	Author year Country	Random sequence generation?	Groups similar at baseline?	Allocation concealed?	Animals randomly housed?	Blinding of caregivers and/or examiners?	Random selection for outcome assessment?	Blinding of outcome assessor?	Incomplete outcome data addressed?	Free from selective outcome reporting?	Free from other bias?
2009	Nemeth et al. (14) United States	U	U	U	U	U	U	U	L	L	L
2010	Bi et al. (15) China	U	U	U	U	U	U	U	H	L	L
2010	Mei et al. (16)A Canada	U	U	U	U	U	U	U	L	L	H
2011	Mei et al. (16)B Canada	U	U	U	U	U	U	U	L	L	L
2012	Liang et al. (17) China	U	U	U	U	U	U	U	U	L	L
2013	Krasnodembskaya et al. (18) USA	U	U	U	U	U	U	U	U	L	L
2013	Hall et al. (19) USA	U	U	U	U	U	U	U	U	L	L
2013	Zhao et al. (20) China	U	U	U	U	U	U	U	U	L	L
2014	Chao et al. (21) Taiwan	U	U	U	U	U	U	U	U	L	L
2014	Kim et al. (22) Canada	U	U	U	U	U	U	H	U	L	L
2014	Luo et al. (23) China	U	U	U	U	U	U	U	U	L	L
2014	Sepulveda et al. (24) Spain	U	U	U	U	U	U	U	U	L	L
2014	Kim et al. (22) Canada	U	U	U	U	U	U	U	U	L	L
2015	Hao Ou et al. (25) China	U	U	U	U	U	U	U	L	L	L
2015	Hao Ou et al. (25) China	U	U	U	U	U	U	U	U	L	L
2015	Güldner et al. (26) Brazil	U	U	U	U	U	U	U	U	L	L
2016	Liu et al. (27) China	U	U	U	U	U	U	U	U	L	L
2017	Xu et al. (28) China	U	U	U	U	U	U	U	U	L	L
2018	Li et al. (29) China	U	U	U	U	U	U	U	U	L	L
2018	Saeedi et al. (30) Iran	U	U	U	U	U	U	U	U	L	L
2018	Saeedi et al. (31) Iran	U	U	U	U	U	U	U	U	L	L
2019	Laroye et al. (32) France	U	U	U	U	U	U	U	H	L	L
2020	Luo et al. (33) China	U	U	U	U	U	U	U	U	L	L
2020	Silva et al. (34) Brazil	U	U	U	U	U	U	U	H	L	L
2021	Varkouhi AK, et al. (35) Canada	U	U	U	U	U	U	U	L	L	L
2021	Guo et al. (36) China	U	U	U	U	U	U	U	U	L	L

H, High risk of bias; L, Low risk of bias; U, Unclear risk of bias.

### Sensitivity Analysis

**Figure 2:**
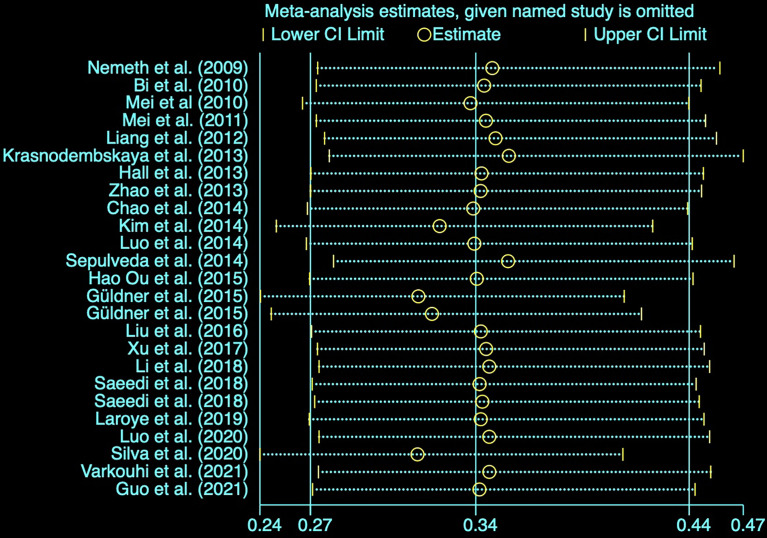
Sensitivity analysis of the studies.

We conducted a sensitivity analysis to evaluate the stability of the results by sequential omission of each study if heterogeneity between the studies existed. The rate of mortality outcome was not significantly affected by any study (**Figure 2**).

### Meta-Analysis

**Figure 3:**
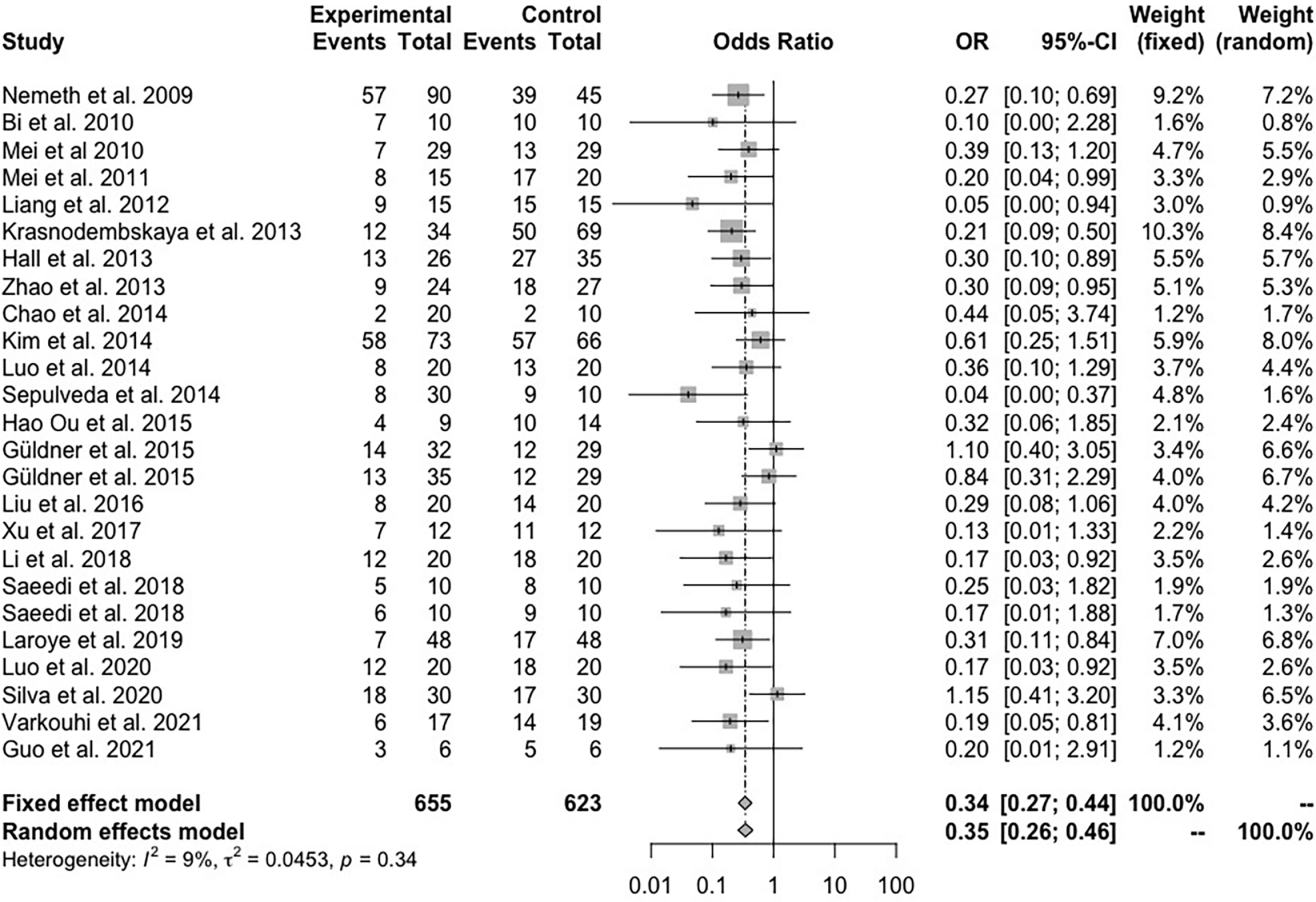
Forest plot summarizing the effects of mesenchymal stem cell therapy on the mortality of preclinical models of sepsis and endotoxemia.

A total of 24 animal studies involving 1278 animals were used in this meta-analysis and reported animal mortality rates. Heterogeneity test results showed *I* ^2^ = 9% and *P* = 0.34, indicating that the heterogeneity between the studies was low; thus, a fixed effects model was used. As shown in **Figure 3**, the pooled results demonstrated that the mortality of the animals after MSC(M) treatment was significantly reduced (OR 0.34, 95% CI 0.27-0.44, *P<*0.0001).

### Stratified Analysis and Meta-Regression Analysis

Subgroup analysis was performed based on the animal species and model, as well as the model gender, species, Sepsis model, and MSC(M) dose, injection time, source, and injection route. In general, significant efficacy of MSC(M) transplantation was observed in most subgroups. In animal models with female participants, MSC(M) for females (OR=0.26, 95% CI 0.13-0.49, P <0.001) had a greater effect than MSC(M) for male participants (OR=0.27, 95% CI 0.19-0.30, P<0.001) and not reported group (OR=0.69, 95% CI 0.42-1.13, P<0.001) (**Figure S1**). Furthermore, MSC(M) administered to non-CLP-induced sepsis animals (OR=0.26, 95% CI 0.16-0.41, P<0.001) were more beneficial than those administered to CLP-induced sepsis animals (OR=0.39, 95% CI 0.28-0.52, P<0.001) (**Figure S2**), and MSC(M) administered to rats (OR=0.23, 95% CI 0.10-0.55, P<0.001) were more beneficial than those administered to mice (OR= 0.36, 95% CI 0.27-0.45, P<0.001) (**Figure S3**). A significant decrease in mortality rate is observed in both doses of ≥1×10^6^ and <1×10^7^ MSC(M) (OR=0.21, 95% CI 0.14-0.32, P<0.001) and ≥1×10^7^ MSC(M) (OR=0.32, 95% CI 0.06-1.85, P<0.001), as compared to doses of <1×10^6^ MSC(M) (OR=0.50, 95% CI 0.35-0.70, P<0.001) (**Figure S4**). Additionally, injection times of ≤6h (OR=0.26, 95% CI 0.19-0.37, P<0.001) and better than >6h (OR=0.48, 95% CI 0.33-0.70, P<0.001) after sepsis induction were noted (**Figure S5**). Further, xenogenic MSC(M) (OR=0.30, 95% CI 0.19-0.47, P<0.001) significantly reduced mortality compared to syngeneic MSC(M) (OR=0.33, 95% CI 0.21-0.51, P < 0.001) and allogenic MSC(M) (OR=0.40, 95% CI 0.20-0.62, P<0.001) (**Figure S6**). As well, intraperitoneal administration (OR=0.04, 95% CI 0.00-0.37, P<0.001) is superior to intravenous administration (OR=0.36, 95% CI 0.28-0.46, P<0.001) after sepsis induction in animal models (**Figure S7**).

### Publication Bias

**Figure 4:**
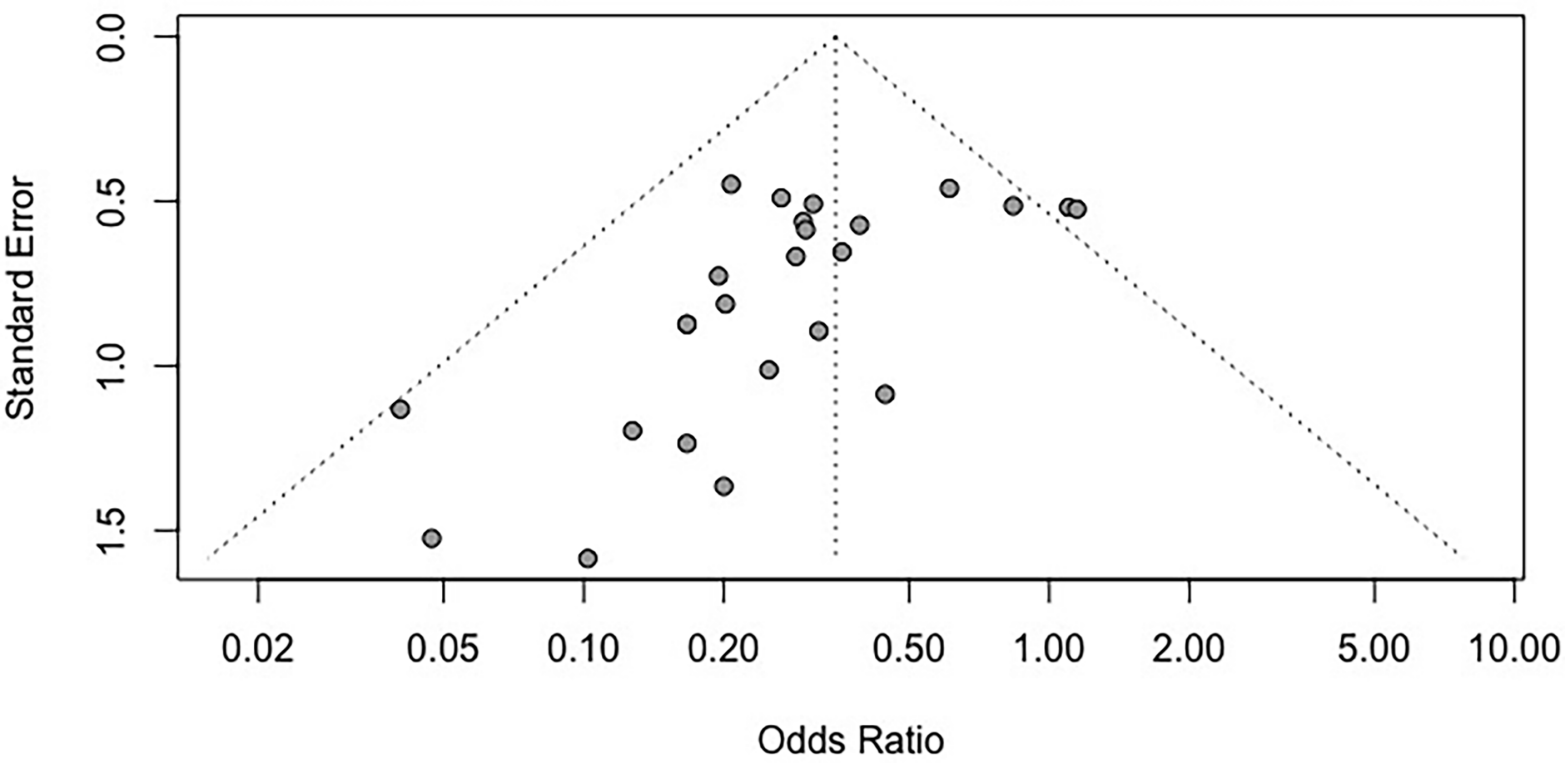
Funnel plot for the mortality rate. Each dot in the figure represents a study, with the y-axis signifying study quality and the x-axis showing the study results. SMD, standardized mean difference.

We assessed publication bias by funnel plots (**Figure 4**). No evident publication bias was observed by visual inspection. The Egger’s test presented publication bias for mortality rates (P = 0.004). After adopting trim-and-fill correction for mortality rates outcomes, the estimated value remained unchanged.

## Discussion

**Figure 5:**
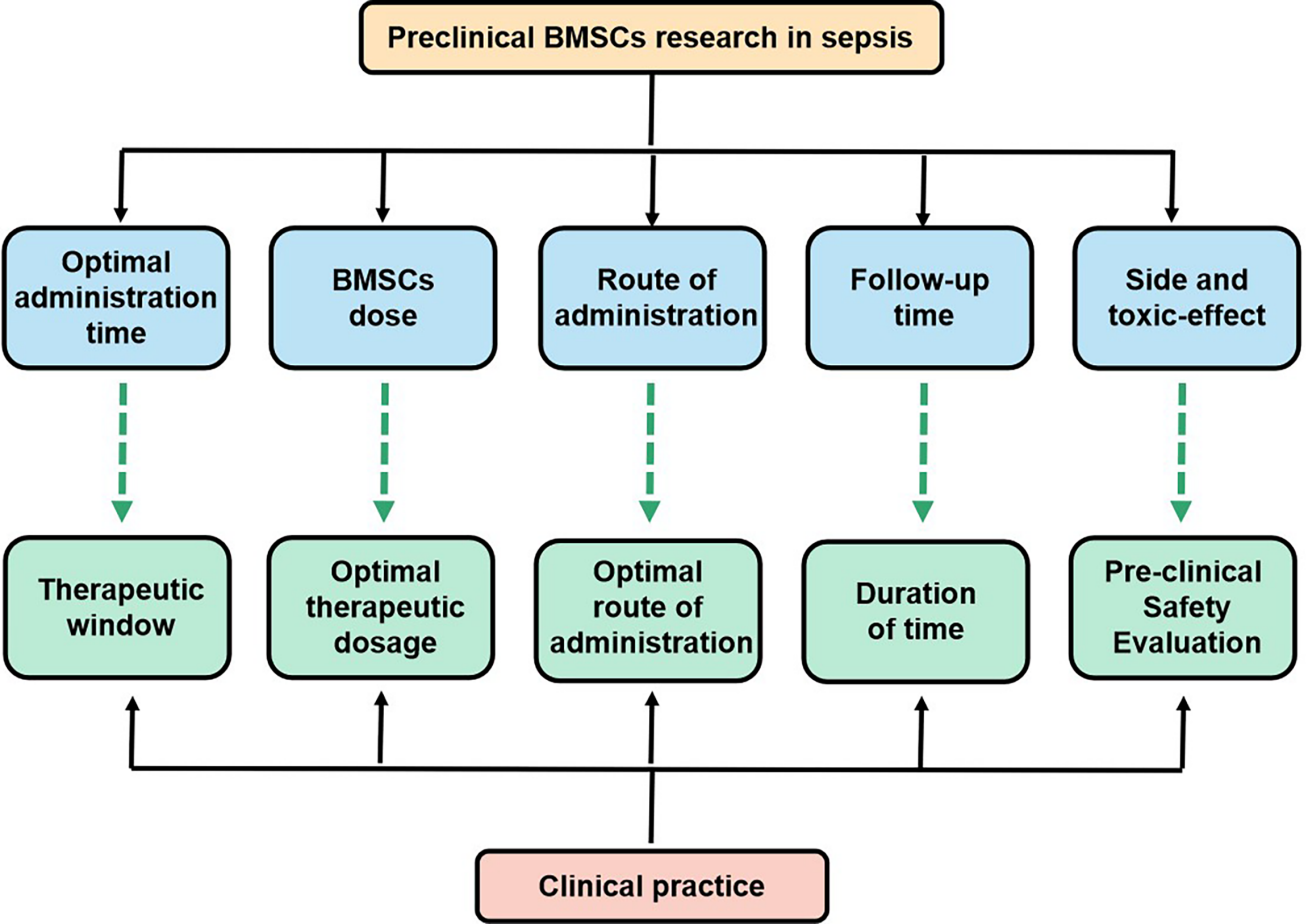
Key issues that the settlement of which would facilitate the transition of BMSCs research in sepsis from bench to bedside.

The use of MSC(M) therapy in sepsis has not been implemented in the clinical management of patients with sepsis despite numerous preclinical studies showing that MSC(M) could decrease the mortality rate of sepsis and improve sepsis. For MSC(M), several issues remain unclear with respect to delivery timing, routes of administration, and dosage. Since animal experiments serve as a basis for designing clinical trials, it is important to examine the combined effects of preclinical and clinical studies. How are stem cell therapies indicated for acute or chronic sepsis, what doses of MSC(M) are optimal, how are MSC(M) delivered, how long do MSC(M) survive in the hostile environment of the disease, and how can sepsis mortality be further improved? (**Figure 5**). It has been reported that previous meta-analyses either considered multiple different mesenchymal stem cell types or that the data was only available until October 1, 2017. However, neither has provided a meta-analysis of the relevant study to examine the mortality rate following MSC(M) transplantation for sepsis. In this study, we demonstrate that MSC(M) can potentially reduce mortality rates of sepsis in animal models. This provides insight into the potential therapeutic applications of MSC(M) in preclinical studies of sepsis. According to our knowledge, two clinical phases 1 trials (**Table 3**) have been conducted to test the safety and feasibility of MSC(M) therapy for patients with septic shock and sepsis. Based on these studies, there were no serious clinical or physiological safety signals, drawing the conclusion that MSC(M) treatment was safe and well-tolerated in critical patients experiencing a septic shock.

**Table 3:** Clinical trials using BMSCs in PD that are registered with ClincalTrials.gov.

No. NCT	Years	Type of Trial	Locations	Recruitment Status	Phase	Ages (Years)	Allo/Auto	Route of Administration	No. of BMSCs	Follow-Up Period
NCT02421484	2015	Open Label	Canada	Completed	Phase 1	18 Years and older	Allogeneic	intravenous	0.3 million cells/kg, 1.0 million cells/kg, and 3.0 million cells/kg	28 months
NCT01849237	2013	Open Label	Russia	Unknown	Phase 1 Phase 2	17 Years to 75 Years	Allogeneic	intravenous	1-2 millions/kg/day	25 months

### Main Findings

According to the results of this meta-analysis: (1) MSC(M) therapy significantly reduced the mortality rate of sepsis animal models, supporting the possibility of using MSC(M) therapy for preclinical studies of sepsis. (2) The administration time was correlated with mortality. MSC(M) therapy initiated within 6 hours of the onset of sepsis exhibited the greatest efficacy. (3) For the mortality rate, intraperitoneal administration injection seems to show the greatest efficacy, followed by intravenous administration, however, this finding should be interpreted cautiously. (4) Xenogenic MSC(M) significantly reduced the mortality rate of sepsis in the animal models, compared to Syngeneic and allogeneic MSC(M). (5) It is unclear from the included studies what is the appropriate dosage of MSC(M). In contrast to the previously published meta-analysis (38), which indicated that less than or equal to 1.0 × 10^6^ MSCs was the ideal dose, the conclusion of our analysis was that more than or equal to 1.0 ×10^6^ MSC(M) is the ideal dose. Therefore, it will be necessary to conduct additional research to determine the ideal dose of MSC(M) for the treatment of sepsis. As a general rule, the above various subgroup analyses are only able to generate hypotheses rather than confirm them. As a result of the subgroup analysis, the results are speculative because they are based on the reanalysis of published data rather than the results of a well-constructed randomized controlled trial. Thus, despite the fact that the subgroup analysis provided updated evidence, the results of this analysis should be considered cautiously.

### Possible Mechanisms of MSC(M) for Sepsis

**Figure 6:**
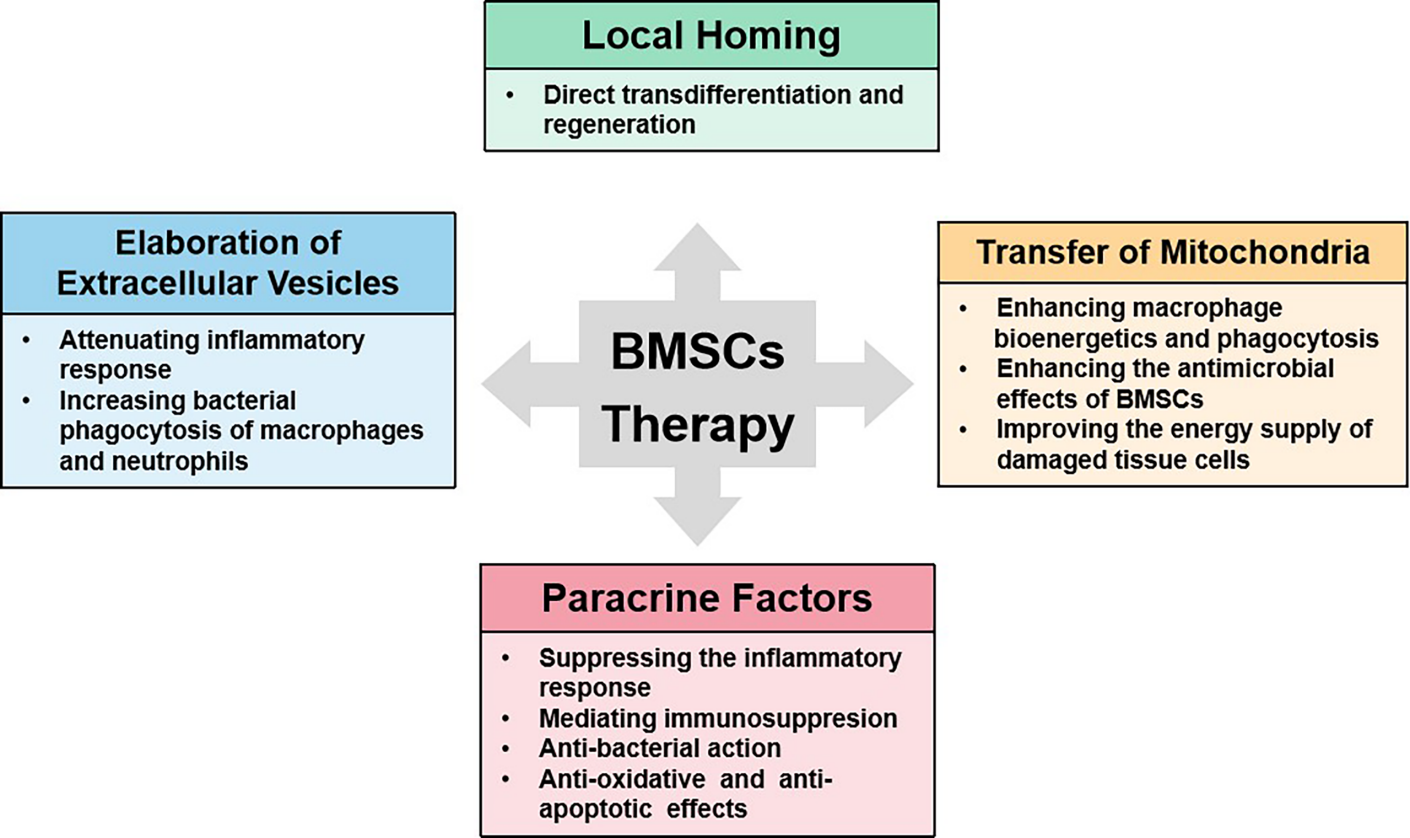
The possible mechanisms of BMSCs therapy for sepsis.

Numerous preclinical studies have demonstrated the benefit of MSC(M) in animal models of sepsis. The precise mechanism by which MSC(M) may exert beneficial effects in sepsis is still being elucidated, but it appears that multiple mechanisms may contribute. Besides mesenchymal stem cells’ homing properties, progress has also been made with understanding the mechanisms of benefit, including paracrine factors, mitochondrial transfer, and the development of extracellular vesicles and microvesicles. (**Figure 6**).

Firstly, MSC(M) may be most helpful in alleviating sepsis due to their anti-inflammatory properties. The MSC(M) can promote the repolarization of monocytes and/or macrophages from a type 1 to a type 2 phenotype, characterized by high levels of intracellular IL-10 secretion, increased phagocytosis, low levels of TNF‐α and interferon‐γ production (49). Reprogrammed type 2 monocytes produced by MSC(M) produce large amounts of IL-10, which inhibits neutrophil influx into injured tissue and prevents further damage. Additionally, MSC(M) can also interfere with the differentiation, maturation, and function of dendritic cells, causing them to adopt a regulatory phenotype (50, 51). The MSC(M) can modulate natural killer cells, which are involved in eradicating virus-infected and damaged cells, as well as releasing a range of proinflammatory cytokines, such as interferon‐γ. It has been demonstrated in a number of studies that MSC(M), in co-culture with natural killer cells, impair their cytotoxic activity, their cytokine production, and their ability to release granzyme B (52). Moreover, MSC(M) have also been shown to have antimicrobial effects in animals. MSC(M) contributed to improved bacterial clearance by secreting antibacterial proteins/peptides such as LL-37 (53),lipocalin-2 (54), and so on. Secondly, several studies have demonstrated that MSC(M) have beneficial effects on metabolomics through mitochondrial transfer using extracellular vesicles. In a model of lipopolysaccharide-induced ALI, mitochondrial transfer through connexin-43 was partially responsible for restoring ATP levels (55). The therapeutic effects of MSC(M) in LPS-induced ALI were abrogated when mutant MSC(M) with defective gap junctions or MSC(M) with dysfunctional mitochondria were used. In addition, a model of E.coli pneumonia has shown that mitochondria transfer from MSC(M) to macrophages is necessary for enhancing macrophage bioenergetics and phagocytosis, as well as the antimicrobial effects of MSC(M) *in vivo (* 56). Finally, extracellular vesicles (EVs) derived from MSC(M) may contribute to the paracrine effect. These MSC(M)-EVs contribute molecules (such as proteins, peptides, mRNA, microRNA, and lipids) with immunoregulatory properties. MSC(M)-EVs have been found to mimic MSC(M) in alleviating sepsis in healthy people and may serve as an alternative to whole-cell therapy. In contrast to MSC(M), MSC(M)-EVs may offer specific advantages due to their low immunogenicity and high safety profile. Even though several studies have demonstrated the benefits of MSC(M)-EVs in sepsis, the underlying mechanisms of MSC(M)-EVs remain unclear. In order to further understand the mechanism by which beneficial effects are mediated, we need to increase our understanding of paracrine factors and the transfer of mitochondria and microcapsules.

### Limitations

We have identified several limitations to our meta-analysis. Firstly, our approach can include only those studies that have been traditionally published in English. Unpublished data may influence our results. Furthermore, none of the included studies examined the safety of MSC(M) injection in sepsis models in animals. We are unable to evaluate the clinical safety of MSC(M). Finally, a good study requires a sample size calculation that is based on formal procedures. However, no study in the meta-analysis had conducted a sample calculation, indicating insufficient statistical power to determine the treatment effect.

## Conclusions and Future Directions

The limited treatment options for sepsis call for new interventions. To date, preclinical studies in rodents have shown MSC(M) to be an effective treatment for sepsis using preclinical research. The study of the efficacy of MSC(M) transplantation for sepsis has identified important future directions that preclinical research needs to address before clinical trials begin. Research gaps identified include the inability to directly compare routes, doses, sources, and timings of MSC(M) administration, as well as the lack of standardization of clinically meaningful behavioral outcomes. The effectiveness of MSC(M) transplantation for sepsis must be evaluated in large animal studies and further investigation is required to determine whether immunosuppression is necessary.

## Data Availability Statement

The original contributions presented in the study are included in the article/**Supplementary Material**. Further inquiries can be directed to the corresponding author.

## Author Contributions

LZ supervised the project. LG, JZ, and HD analyzed the data. ZH and CC extracted the data. LG and LZ wrote the paper. All authors contributed to the article and approved the submitted version.

## Funding

This work was supported by National Natural Science Foundation of China (No. 31670838, No.32000151, No.81371358, No.81974213), Key Project of Hunan Provincial Science and Technology Innovation (2020SK2102), Hunan Provincial Natural Science Foundation of China (2021JJ40830).

## Conflict of Interest

The authors declare that the research was conducted in the absence of any commercial or financial relationships that could be construed as a potential conflict of interest.

## Publisher’s Note

All claims expressed in this article are solely those of the authors and do not necessarily represent those of their affiliated organizations, or those of the publisher, the editors and the reviewers. Any product that may be evaluated in this article, or claim that may be made by its manufacturer, is not guaranteed or endorsed by the publisher.
