# Supplementary material for: Effect of Bone Marrow Mesenchymal Stromal Cell Therapies in Rodent Models of Sepsis: A Meta-Analysis
Source: Front Immunol. 2022 Jan 3;12:792098. doi: 10.3389/fimmu.2021.792098 (PMC8761857; doi:10.3389/fimmu.2021.792098)
Supplement: Supplementary file 1 [file DataSheet_1.doc]

**Supplement materials**

Additional file 1: The detailed search strategy.

**The Detailed Search Strategy**

**Pubmed：**

 (("Sepsis"[Mesh]) OR (((((((((((((((((Bloodstream Infection[Title/Abstract]) OR (Bloodstream Infections[Title/Abstract])) OR (Infection, Bloodstream[Title/Abstract])) OR (Pyemia[Title/Abstract])) OR (Pyemias[Title/Abstract])) OR (Pyohemia[Title/Abstract])) OR (Pyohemias[Title/Abstract])) OR (Pyaemia[Title/Abstract])) OR (Pyaemias[Title/Abstract])) OR (Septicemia[Title/Abstract])) OR (Septicemias[Title/Abstract])) OR (Poisoning, Blood[Title/Abstract])) OR (Blood Poisoning[Title/Abstract])) OR (Blood Poisonings[Title/Abstract])) OR (Poisonings, Blood[Title/Abstract])) OR (Severe Sepsis[Title/Abstract])) OR (Sepsis, Severe[Title/Abstract]))) AND (((Stem Cell, Mesenchymal[Title/Abstract]) OR (Stem Cells, Mesenchymal[Title/Abstract]) OR (Mesenchymal Stem Cell[Title/Abstract]) OR (Bone Marrow Mesenchymal Stem Cells[Title/Abstract]) OR (Bone Marrow Mesenchymal Stem Cell[Title/Abstract]) OR (Bone Marrow Stromal Cells[Title/Abstract]) OR (Bone Marrow Stromal Cell[Title/Abstract]) OR (Bone Marrow Stromal Cells, Multipotent[Title/Abstract]) OR (Multipotent Bone Marrow Stromal Cell[Title/Abstract]) OR (Multipotent Bone Marrow Stromal Cells[Title/Abstract]) OR (Adipose-Derived Mesenchymal Stem Cells[Title/Abstract]) OR (Adipose Derived Mesenchymal Stem Cells[Title/Abstract]) OR (Adipose Tissue-Derived Mesenchymal Stem Cell[Title/Abstract]) OR (Adipose Tissue Derived Mesenchymal Stem Cell[Title/Abstract]) OR (Adipose Tissue-Derived Mesenchymal Stem Cells[Title/Abstract]) OR (Adipose Tissue Derived Mesenchymal Stem Cells[Title/Abstract]) OR (Mesenchymal Stem Cells, Adipose-Derived[Title/Abstract]) OR (Mesenchymal Stem Cells, Adipose Derived[Title/Abstract]) OR (Adipose Tissue-Derived Mesenchymal Stromal Cells[Title/Abstract]) OR (Adipose Tissue Derived Mesenchymal Stromal Cells[Title/Abstract]) OR (Adipose-Derived Mesenchymal Stromal Cells[Title/Abstract]) OR (Adipose Derived Mesenchymal Stromal Cells[Title/Abstract]) OR (Adipose-Derived Mesenchymal Stem Cell[Title/Abstract]) OR (Adipose Derived Mesenchymal Stem Cell[Title/Abstract]) OR (Mesenchymal Stromal Cells[Title/Abstract]) OR (Stromal Cell, Mesenchymal[Title/Abstract]) OR (Stromal Cells, Mesenchymal[Title/Abstract]) OR (Mesenchymal Stromal Cell[Title/Abstract]) OR (Multipotent Mesenchymal Stromal Cells[Title/Abstract]) OR (Mesenchymal Stromal Cells, Multipotent[Title/Abstract]) OR (Multipotent Mesenchymal Stromal Cell[Title/Abstract]) OR (Mesenchymal Progenitor Cell[Title/Abstract]) OR (Mesenchymal Progenitor Cells[Title/Abstract]) OR (Progenitor Cell, Mesenchymal[Title/Abstract]) OR (Progenitor Cells, Mesenchymal[Title/Abstract]) OR (Wharton Jelly Cells[Title/Abstract]) OR (Wharton's Jelly Cells[Title/Abstract]) OR (Wharton's Jelly Cell[Title/Abstract]) OR (Whartons Jelly Cells[Title/Abstract]) OR (Bone Marrow Stromal Stem Cells[Title/Abstract])) OR ("Mesenchymal Stem Cells"[Mesh]))=184 articles

**Web of science：**

#1 TS=(Mesenchymal Stem Cells OR Stem Cell, Mesenchymal OR Stem Cells, Mesenchymal OR Mesenchymal Stem Cell OR Bone Marrow Mesenchymal Stem Cells OR Bone Marrow Mesenchymal Stem Cell OR Bone Marrow Stromal Cells OR Bone Marrow Stromal Cell OR Bone Marrow Stromal Cells, Multipotent OR Multipotent Bone Marrow Stromal Cell OR Multipotent Bone Marrow Stromal Cells OR Adipose-Derived Mesenchymal Stem Cells OR Adipose Derived Mesenchymal Stem Cells OR Adipose Tissue-Derived Mesenchymal Stem Cell OR Adipose Tissue Derived Mesenchymal Stem Cell OR Adipose Tissue-Derived Mesenchymal Stem Cells OR Adipose Tissue Derived Mesenchymal Stem Cells OR Mesenchymal Stem Cells, Adipose-Derived OR Mesenchymal Stem Cells, Adipose Derived OR Adipose Tissue-Derived Mesenchymal Stromal Cells OR Adipose Tissue Derived Mesenchymal Stromal Cells OR Adipose-Derived Mesenchymal Stromal Cells OR Adipose Derived Mesenchymal Stromal Cells OR Adipose-Derived Mesenchymal Stem Cell OR Adipose Derived Mesenchymal Stem Cell OR Mesenchymal Stromal Cells OR Stromal Cell, Mesenchymal OR Stromal Cells, Mesenchymal OR Mesenchymal Stromal Cell OR Multipotent Mesenchymal Stromal Cells OR Mesenchymal Stromal Cells, Multipotent OR Multipotent Mesenchymal Stromal Cell OR Mesenchymal Progenitor Cell OR Mesenchymal Progenitor Cells OR Progenitor Cell, Mesenchymal OR Progenitor Cells, Mesenchymal OR Wharton Jelly Cells OR Wharton's Jelly Cells OR Wharton's Jelly Cell OR wharton Jelly Cells OR Bone Marrow Stromal Stem Cells )  163,406 articles

#2 TS=(Sepsis OR Bloodstream Infection OR Bloodstream Infections OR Infection, Bloodstream OR Pyemia OR Pyemias OR Pyohemia OR Pyohemias OR Pyaemia OR Pyaemias OR Septicemia OR Septicemias OR Poisoning, Blood OR Blood Poisoning OR Blood Poisonings OR Poisonings, Blood OR Severe Sepsis OR Sepsis, Severe )

350939 articles

#3=#1 AND #2 784 articles

**EMBASE:**

#1 'mesenchymal stem cell'/exp results：68,013 articles

#2 'stem cell, mesenchymal':ab,ti OR 'stem cells, mesenchymal':ab,ti OR 'mesenchymal stem cell':ab,ti OR 'bone marrow mesenchymal stem cells':ab,ti OR 'bone marrow mesenchymal stem cell':ab,ti OR 'bone marrow stromal cells':ab,ti OR 'bone marrow stromal cell':ab,ti OR 'bone marrow stromal cells, multipotent':ab,ti OR 'multipotent bone marrow stromal cell':ab,ti OR 'multipotent bone marrow stromal cells':ab,ti OR 'adipose-derived mesenchymal stem cells':ab,ti OR 'adipose derived mesenchymal stem cells':ab,ti OR 'adipose tissue-derived mesenchymal stem cell':ab,ti OR 'adipose tissue derived mesenchymal stem cell':ab,ti OR 'adipose tissue-derived mesenchymal stem cells':ab,ti OR 'adipose tissue derived mesenchymal stem cells':ab,ti OR 'mesenchymal stem cells, adipose-derived':ab,ti OR 'mesenchymal stem cells, adipose derived':ab,ti OR 'adipose tissue-derived mesenchymal stromal cells':ab,ti OR 'adipose tissue derived mesenchymal stromal cells':ab,ti OR 'adipose-derived mesenchymal stromal cells':ab,ti OR 'adipose derived mesenchymal stromal cells':ab,ti OR 'adipose-derived mesenchymal stem cell':ab,ti OR 'adipose derived mesenchymal stem cell':ab,ti OR 'mesenchymal stromal cells':ab,ti OR 'stromal cell, mesenchymal':ab,ti OR 'stromal cells, mesenchymal':ab,ti OR 'mesenchymal stromal cell':ab,ti OR 'multipotent mesenchymal stromal cells':ab,ti OR 'mesenchymal stromal cells, multipotent':ab,ti OR 'multipotent mesenchymal stromal cell':ab,ti OR 'mesenchymal progenitor cell':ab,ti OR 'mesenchymal progenitor cells':ab,ti OR 'progenitor cell, mesenchymal':ab,ti OR 'progenitor cells, mesenchymal':ab,ti OR 'wharton jelly cells':ab,ti OR 'wharton jelly cell':ab,ti OR 'whartons jelly cells':ab,ti OR 'bone marrow stromal stem cells':ab,ti 46858 articles

#3 = #1 OR #2 91983 articles

#4 'sepsis'/exp 299414 articles

#5 'bloodstream infection':ab,ti OR 'bloodstream infections':ab,ti OR 'infection, bloodstream':ab,ti OR 'pyemia':ab,ti OR 'pyemias':ab,ti OR 'pyohemia':ab,ti OR 'pyohemias':ab,ti OR 'pyaemia':ab,ti OR 'pyaemias':ab,ti OR 'septicemia':ab,ti OR 'septicemias':ab,ti OR 'poisoning, blood':ab,ti OR 'blood poisoning':ab,ti OR 'blood poisonings':ab,ti OR 'poisonings, blood':ab,ti OR 'severe sepsis':ab,ti OR 'sepsis, severe':ab,ti 48052 articles

#6= #4 OR #5 316161 articles

#7=#3 AND #6 538 articles


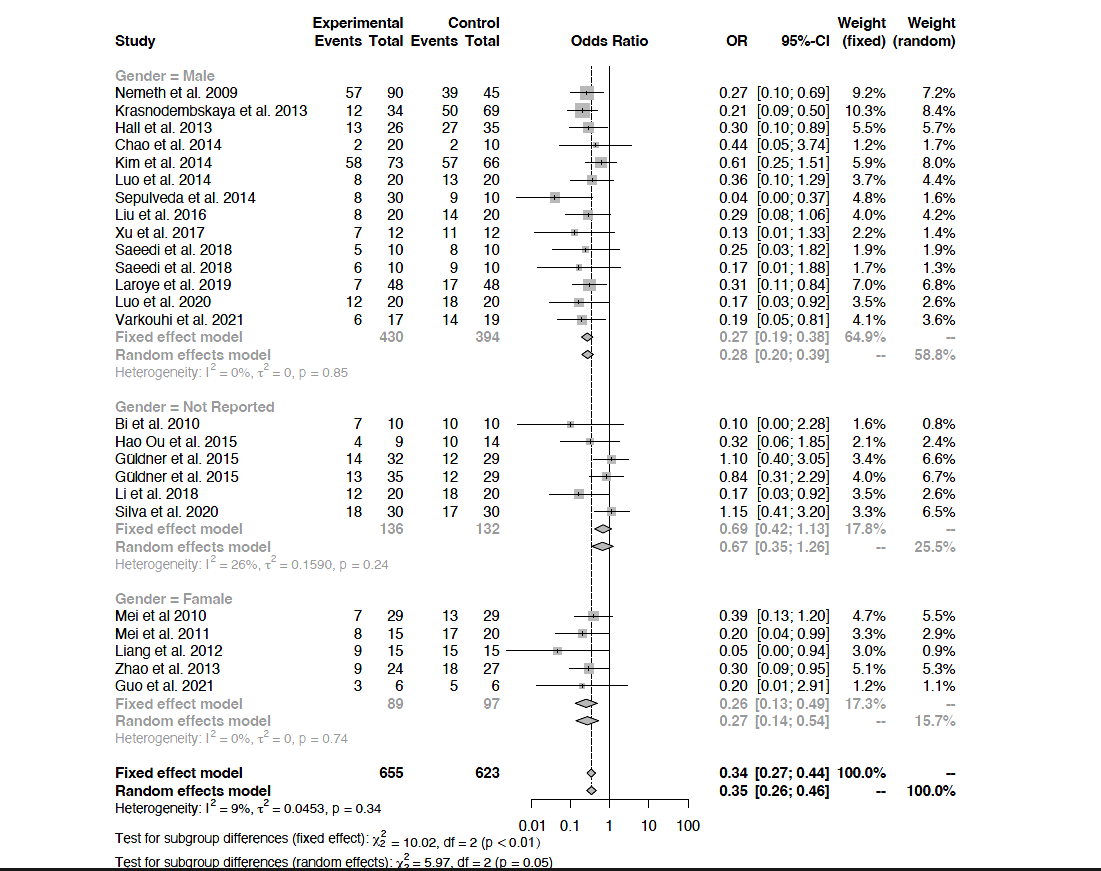


Additional file 2: Fig. S1. Forest plot summarizing the relationship between animal gender and mortality in preclinical models of sepsis.


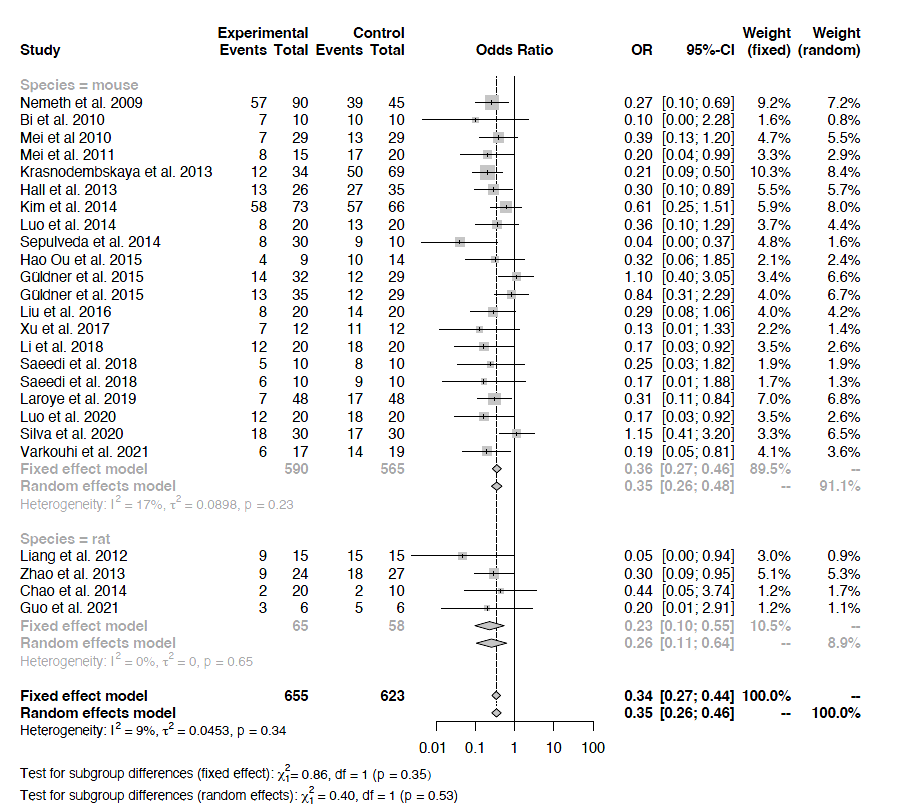


Additional file 3: Fig. S2. Forest plot summarizing the relationship between MSC(M)-treated animal model species (rat versus mouse) and mortality in preclinical models of sepsis.


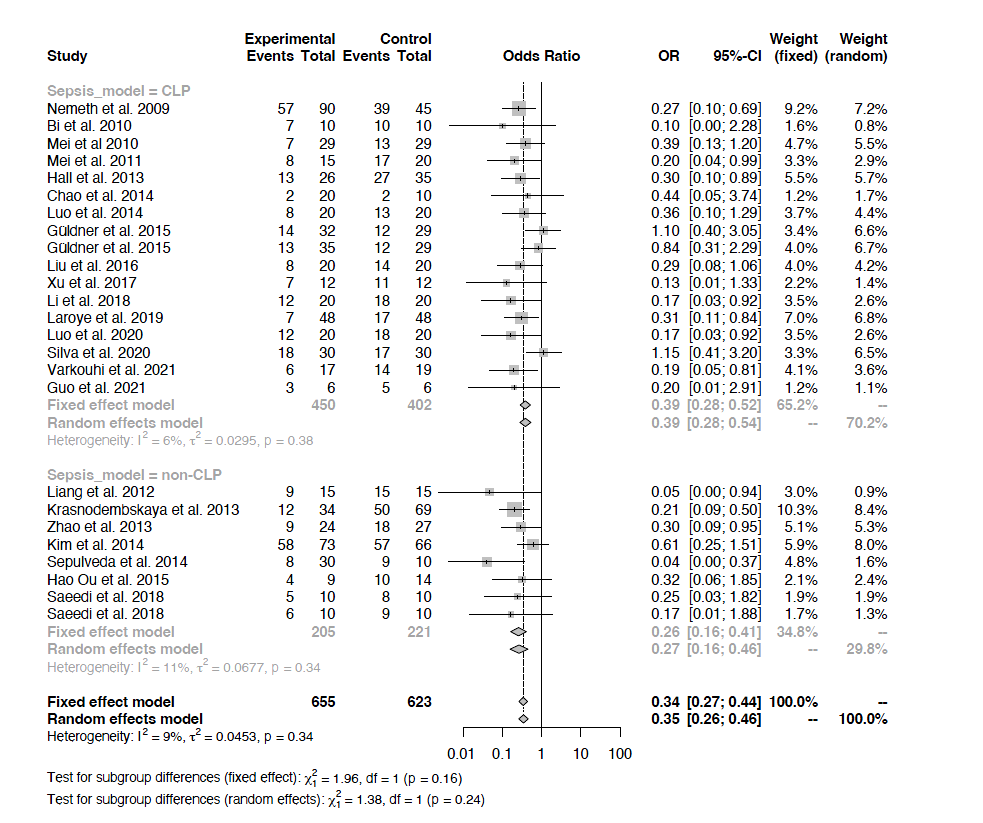


Additional file 4: Fig. S3. Forest plot summarizing the relationship between preclinical models of sepsis (CLP versus non-CLP) and mortality following treatment with mesenchymal stem cells.


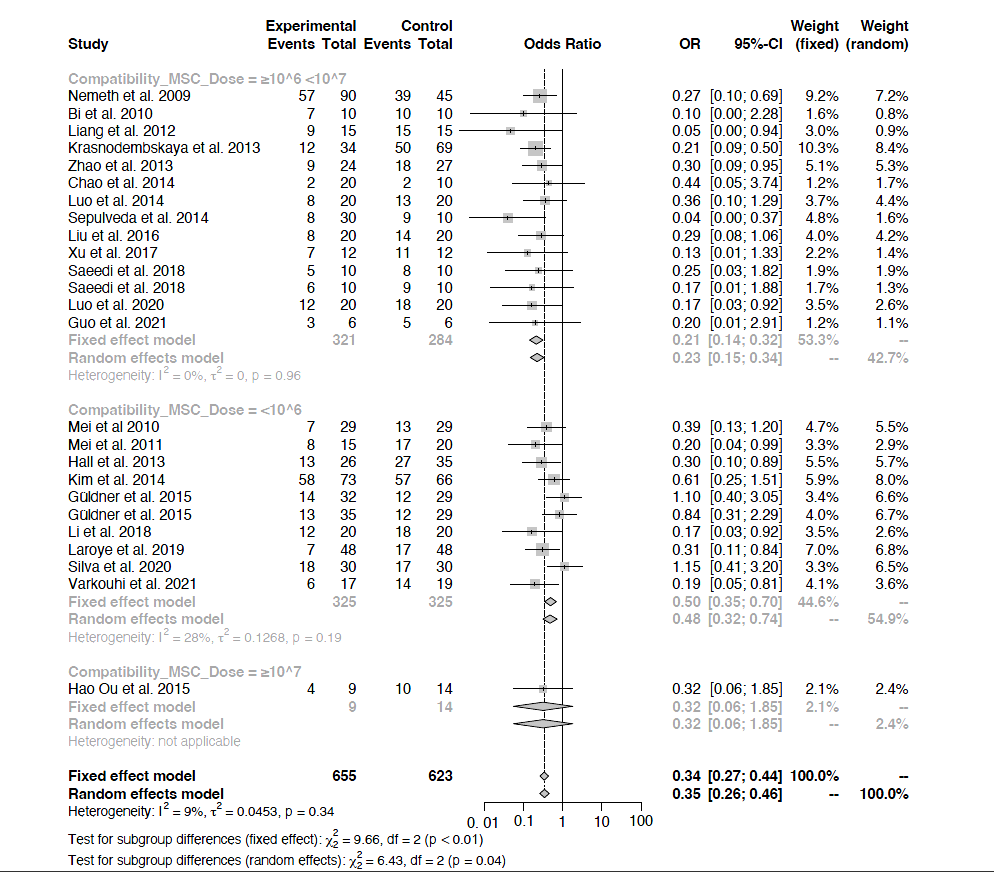


Additional file 5: Fig. S4. Forest plot summarizing the relationship between MSC(M) dose and mortality in preclinical models of sepsis.


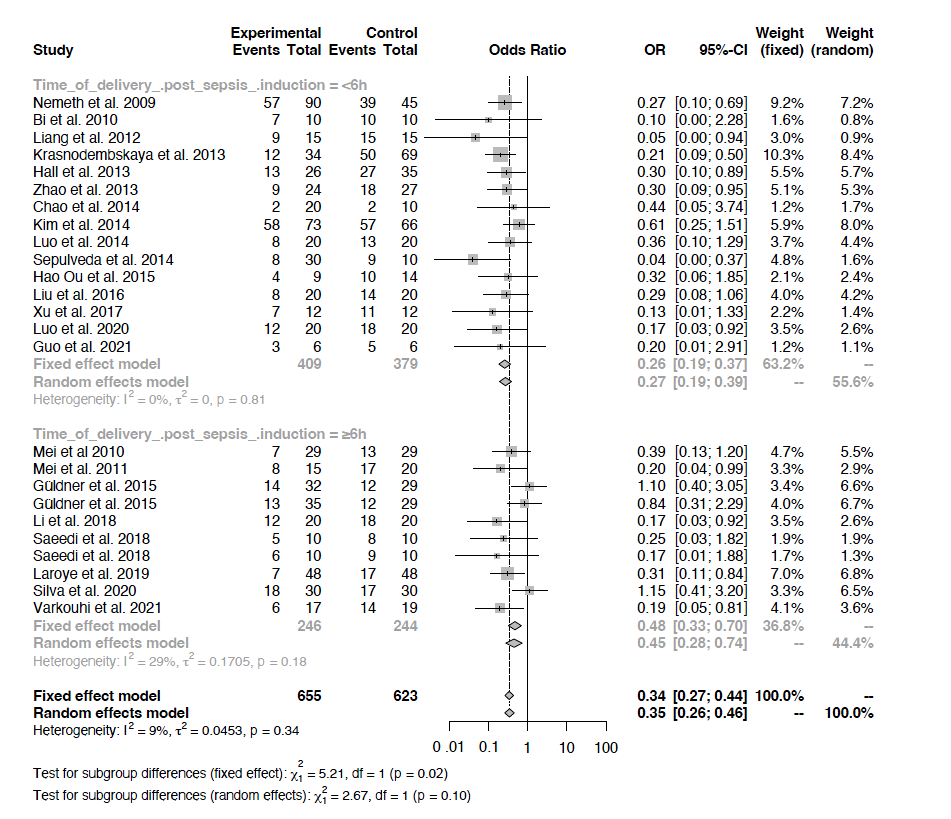


Additional file 6: Fig. S5. Forest plot summarizing the relationship between MSC(M) therapy timing of administration and mortality in preclinical models of sepsis.


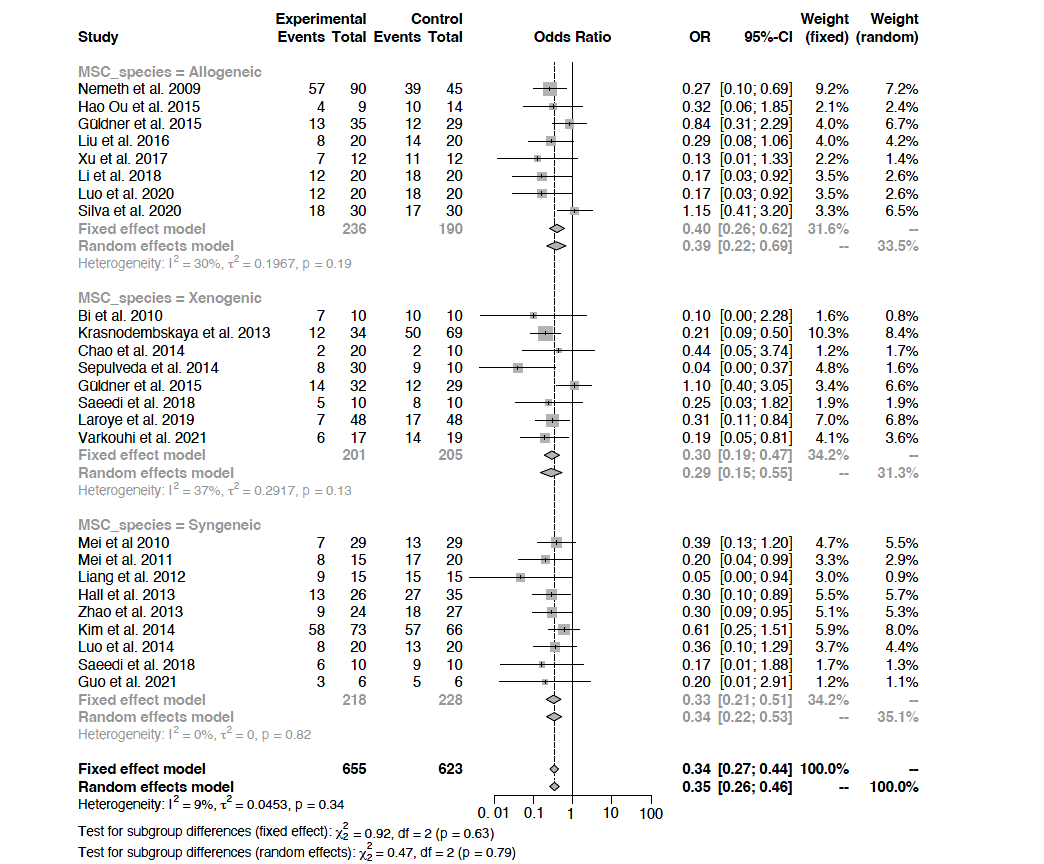


Additional file 7: Fig. S6. Forest plot summarizing the relationship between MSC(M) species (i.e. allogeneic, xenogenic, and syngeneic) and mortality in preclinical models of sepsis.


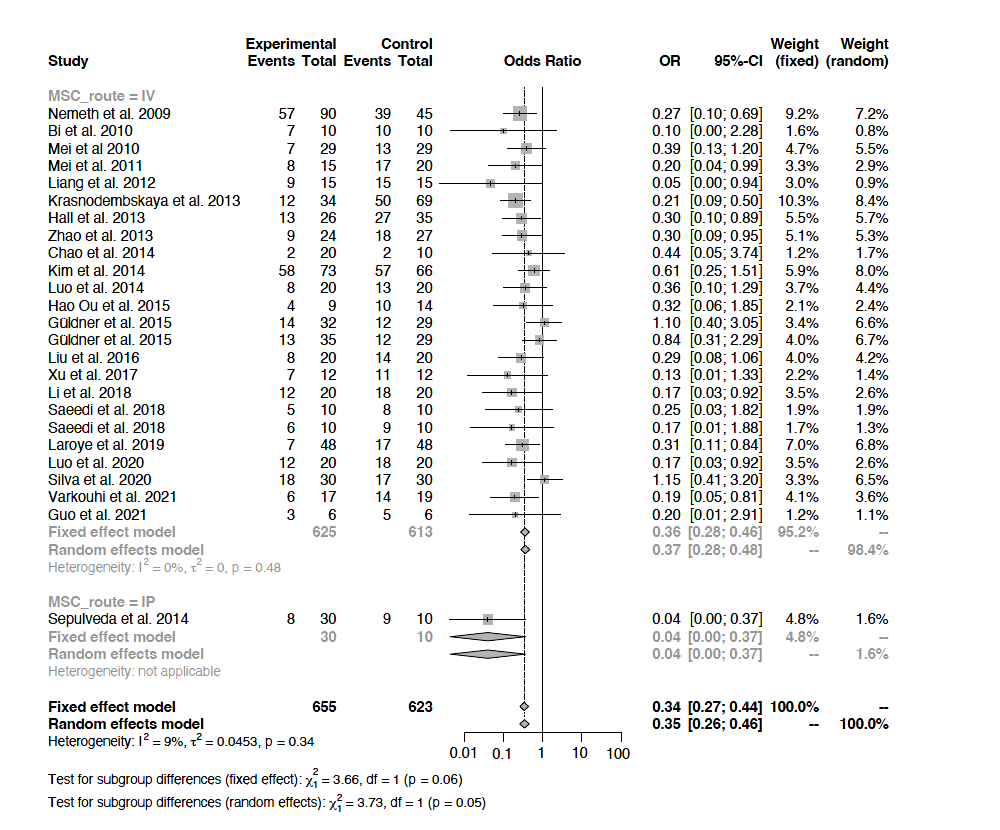
Additional file 8: Fig. S7. Forest plot summarizing the relationship between MSC(M) administration route (intravenous versus intraperitoneal injection) and mortality in preclinical models of sepsis.
